# Supplementary material for: DOT: a flexible multi-objective optimization framework for transferring features across single-cell and spatial omics
Source: Nat Commun. 2024 Jun 11;15:4994. doi: 10.1038/s41467-024-48868-z (PMC11167014; doi:10.1038/s41467-024-48868-z)
Supplement: Supplementary file 1 — Supplementary Information [file 41467_2024_48868_MOESM1_ESM.pdf]

Supplementary Information

DOT: A flexible multi-objective optimization framework for transferring features across single-cell and spatial omics

Arezou Rahimi, Luis Vale Silva, Maria Fälth Savitski, Jovan Tanevski, and Julio Saez-Rodriguez

---

Supplementary Methods

Implementation details of the FW algorithm

Convergence

Under suitable conditions, FW converges to an optimal solution in linear rate when optimizing a convex function over a polytope domain [1]. Given the non-convex objective function in Eq. (6), Algorithm 1 instead obtains a first-order stationary point at a rate of $O(1/\sqrt{t})$ [2, 3]. We numerically assess the convergence of Algorithm 1 at iteration t using the so-called "FW-gap" [4]

$$\delta^{(t)} := \sum_{i \in \mathbb{I}} \sum_{c \in \mathbb{C}} (Y^{(t)}_{c,i} - \hat{Y}^{(t)}_{c,i}) \Delta^{(t)}_{c,i}.$$

We also implemented acceleration techniques such as averaging gradients [5], away steps [1, 6], and entropic regularization but did not observe substantial gains compared to our current implementation of FW.

Initial solution

A good quality initial solution can enhance convergence of FW. Given the multi-objective nature of our model, we produce an initial solution as convex combination of three solutions. In the first solution, for each spot i we first find cell type $\hat{c} = \arg\min_{c \in \mathbb{C}} \{ \mathrm{d}_{\cos} \left(\mathbf{X}^{\mathrm{S}}_{i,:}, \mathbf{X}^{\mathrm{R}}_{c,:} \right) \}$ and set $Y_{c,i} = n_i$ if $c = \hat{c}$ and $Y_{c,i} = 0$ otherwise. Note that this solution is optimal for the sparse case when d_i is the only objective.

We derive the second solution with the goal of optimizing d_g as the sole objective function. Assuming that both $\mathbf{X}^{\mathrm{S}}$ and $\mathbf{X}^{\mathrm{R}}$ are count matrices, we can approximate minimizing d_g by solving a non-negative least squares

$$\min_{\mathbf{Y} \geq \mathbf{0}} \|\mathbf{Y}^\top \mathbf{X}^{\mathrm{R}} - \mathbf{X}^{\mathrm{S}}\|_2^2.$$

To derive a fast solution, we note that all entries of $\mathbf{X}^{\mathrm{S}}$ and $\mathbf{X}^{\mathrm{R}}$ are non-negative. Therefore, a generalized linear regression with the non-negativity constraints relaxed yields a solution $\mathbf{Y}$ in which $Y_{c,i} > 0$ for at least one c for each i. Finally, adding a ridge penalty to account for the cases when $\mathbf{X}^{\mathrm{R}}$ is not full-rank (which typically happens when the number of genes is less than the number of cell populations), we obtain the solution

$$\mathbf{Y} = \left(\mathbf{X}^{\mathrm{R}}\mathbf{X}^{\mathrm{R}\top} + \mathbf{I}_{|C|}\right)^{-1}\mathbf{X}^{\mathrm{R}}\mathbf{X}^{\mathrm{S}\top}, \tag{S1}$$

and set the negative entries of $\mathbf{Y}$ to 0. Given that $|\mathbb{C}|$ is typically small, the matrix inversion in Eq. (S1) can be done easily. Moreover, given that $\mathbf{X}^{\mathrm{S}}$ and $\mathbf{X}^{\mathrm{R}}$ are count matrices, $\sum_c \sum_i Y_{c,i}$ gives an estimate on the total number of cells that can fit in S.

In the third solution, we simply set $Y_{c,i} = \frac{r_c}{\sum_{c'} r_{c'}} n$ for each i and c. Note that this solution is optimal for $\mathrm{d_A}$. We then set the initial solution as the convex combination of these three solutions with weights 0.4, 0.4, 0.2, respectively.

Derivatives

To find the derivatives of $\mathrm{d}_i(\mathbf{Y})$ and $\mathrm{d}_c(\mathbf{Y})$, defined in Eq. (1) and Eq. (2), we introduce auxiliary quantities $\bar{\mathbf{X}}^{\mathrm{S}} \coloneqq \mathbf{Y}^\top\mathbf{X}^{\mathrm{R}}$ and $\bar{\mathbf{X}}^{\mathrm{R}} \coloneqq \mathbf{Y}\mathbf{X}^{\mathrm{S}}$ to denote the expressions transferred through $\mathbf{Y}$ to spots and cell populations, respectively. Derivatives for $\mathrm{d}_i(\mathbf{Y})$ and $\mathrm{d}_c(\mathbf{Y})$ can then be calculated as:

$$\frac{\partial \mathrm{d}_i}{\partial Y_{c,i}} = \frac{1}{\|\mathbf{X}^{\mathrm{S}}_{i,:}\|}\langle \mathbf{X}^{\mathrm{R}}_{c,:}, \mathbf{T}^{\mathrm{S}}_{i,:}\rangle, \qquad \frac{\partial \mathrm{d}_c}{\partial Y_{c,i}} = \frac{1}{\|\mathbf{X}^{\mathrm{R}}_{c,:}\|}\langle \mathbf{X}^{\mathrm{S}}_{i,:}, \mathbf{T}^{\mathrm{R}}_{c,:}\rangle,$$

where

$$\begin{aligned} T^{\mathrm{S}}_{i,g} =& \frac{-1}{2\mathrm{d}_i(\mathbf{Y})}\left(\frac{X^{\mathrm{S}}_{i,g}}{\|\bar{\mathbf{X}}^{\mathrm{S}}_{i,:}\|} - \frac{\bar{X}^{\mathrm{S}}_{i,g}}{\|\bar{\mathbf{X}}^{\mathrm{S}}_{i,:}\|^3}\langle \mathbf{X}^{\mathrm{S}}_{i,:}, \bar{\mathbf{X}}^{\mathrm{S}}_{i,:}\rangle\right), \\ T^{\mathrm{R}}_{c,g} =& \frac{-1}{2\mathrm{d}_c(\mathbf{Y})}\left(\frac{X^{\mathrm{R}}_{c,g}}{\|\bar{\mathbf{X}}^{\mathrm{R}}_{c,:}\|} - \frac{\bar{X}^{\mathrm{R}}_{c,g}}{\|\bar{\mathbf{X}}^{\mathrm{R}}_{c,:}\|^3}\langle \mathbf{X}^{\mathrm{R}}_{c,:}, \bar{\mathbf{X}}^{\mathrm{R}}_{c,:}\rangle\right). \end{aligned}$$

Similarly, we may derive the derivatives for $\mathrm{d}_g(\mathbf{Y})$ defined in Eq. (3) via

$$\frac{\partial \mathrm{d}_g}{\partial Y_{c,i}} = \frac{-1}{2\mathrm{d}_g(\mathbf{Y})}\frac{X^{\mathrm{R}}_{c,g}}{\|\mathbf{X}^{\mathrm{S}}_{:,g}\|}\left(\frac{X^{\mathrm{S}}_{i,g}}{\|\bar{\mathbf{X}}^{\mathrm{S}}_{:,g}\|} - \frac{Y_{c,i}}{\|\bar{\mathbf{X}}^{\mathrm{S}}_{:,g}\|^3}\langle \mathbf{X}^{\mathrm{S}}_{:,g}, \bar{\mathbf{X}}^{\mathrm{S}}_{:,g}\rangle\right)$$

The derivatives for $\mathrm{d_S}$ defined in Eq. (4) can be computed as

$$\frac{\partial \mathrm{d_S}}{\partial Y_{c,i}} = \frac{1}{2} \sum_{j \in \mathbb{I}:(i,j) \in \mathbb{P} \text{ or } (j,i) \in \mathbb{P}} \log \left(\frac{2Y_{c,i}}{Y_{c,i} + Y_{c,j}} \right).$$

Finally, the derivatives for $\mathrm{d_A}$ defined in Eq. (5) can be calculated as:

$$\frac{\partial \mathrm{d_A}}{\partial Y_{c,i}} = \frac{1}{2} \log \left(\frac{2\rho_c}{\rho_c + r_c} \right).$$

Connection to fused Gromov-Wasserstein Optimal Transport

As discussed in the main body of the paper, our formulation can be viewed as a generalization of Optimal Transport (OT), which is a way to match, with minimal cost, data points between two domains embedded in possibly different spaces using different variants of the Wasserstein distance [7–10]. Over the past years, OT has been applied to various machine learning problems in a wide variety of contexts such as generative modeling [11], feature aggregation [12], dataset denoising [13], generalization error prediction [14], graph matching/classification [15], and domain adaptation [16]. In particular, OT has been employed in computational biology with applications such as transporting entities from one cross sectional measurement to the next using unbalanced dynamic transport [17], studying developmental time courses and understanding the molecular programs that guide differentiation during development [18], reconstructing developmental trajectories from time courses with snapshots of cell states and lineages [19], reconstructing the organization of cells in the tissue [20, 21] and alignment of spatial omics [22]. In addition, computational pipelines with OT components have been developed to facilitate applications of OT in computational biology [23].

Here, we elaborate on connections between our formulation and standard OT formulations and highlight the distinct features of our model that separate our formulation from them. An OT formulation in its most basic form for assigning cell populations to spatial locations can be expressed as the following optimization problem:

$$\min_{\mathbf{Z} \geq \mathbf{0}} \quad \sum_{c \in \mathbb{C}} \sum_{i \in \mathbb{I}} C_{c,i} Z_{c,i} \tag{S2}$$

$$\text{s.t.} \quad \sum_{c \in \mathbb{C}} Z_{c,i} = p_i \qquad \forall i \in \mathbb{I} \tag{S3}$$

$$\sum_{i \in \mathbb{I}} Z_{c,i} = q_c \qquad \forall c \in \mathbb{C}, \tag{S4}$$

where $\mathbf{p}$ and $\mathbf{q}$ are given marginal distributions for cell populations and spots, respectively, and $\mathbf{C}$ is the transportation cost matrix which can be computed as the dissimilarity between expression profile of cell populations in R and spots in S.

We first note that the linear cost function in Eq. (S2) is akin to our location-wise cost function d_i in the sparse case when $C_{c,i} = \mathrm{d}_{\cos}(\mathbf{X}^{\mathrm{R}}_{c,:}, \mathbf{X}^{\mathrm{S}}_{i,:})$. More precisely, $\mathrm{d}_i(\mathbf{Z}) = \sum_{c \in \mathbb{C}} C_{c,i} Z_{c,i}$ when the sparsity parameter θ in Eq. (12) is set to 1. However, there are major differences between d_i and the linear cost function which make our distance function d_i more suitable for the task at hand:

(i) First, note that $C_{c,i}$ is computed by assuming that all of location i is occupied by a single cell population c. Therefore, a linear cost function cannot capture the low resolution case as spots in the low-resolution SRT are comprised of multiple cells that potentially belong to different cell populations.

(ii) The second difference between d_i and the linear cost function is that d_i is indifferent to the size of spots in the low-resolution case thanks to the scale invariance property of our $\mathrm{d}_{\cos}$ distance function. In contrast, the linear cost function pushes the size of all spots to the lower limit. More precisely, if we relax Eq. (S4) and replace Eq. (S3) with a two-sided bounded constraint $1 \leq \sum_{c \in \mathbb{C}} Z_{c,i} \leq n_i$, then $\sum_{c \in \mathbb{C}} Z_{c,i} = 1$ at any optimal solution. This means a standard OT formulation (even a partially unbalanced Fused Gromov-Wasserstein formulation; see below) cannot distinguish between the size of different spots.

(iii) Finally, when reliable information about the abundance of cell populations is not available, even a partially unbalanced OT formulation may not be appropriate and the OT formulation results in a trivial solution in which each spot gets assigned to its closest cell population independently of other spots. Note that our centroid distance function d_c and gene map distance function d_g defined in Eq. (2) and Eq. (3), respectively, prevent such a trivial solution even when no prior information about the abundance of cell populations is available.

The second link between our formulation and variants of OT can be characterized via the Fused Gromov-Wasserstein (FGW) formulation, a variant of OT for matching structured data. In our application, given $\mathbf{M}^{\mathrm{R}}$ and $\mathbf{M}^{\mathrm{S}}$ as metrics in the space of R and S, which denote the pairwise dissimilarity between elements of R and S, respectively, FGW combines the linear cost $\sum_{c \in \mathbb{C}} \sum_{i \in \mathbb{I}} C_{c,i} Z_{c,i}$ with the *2-Gromov-Wasserstein*

distance [24] and replaces the objective function in Eq. (S2) with

$$\alpha \sum_{c\in\mathbb{C}} \sum_{i\in\mathbb{I}} C_{c,i}^2 Z_{c,i} + (1-\alpha) \sum_{c\in\mathbb{C}} \sum_{k\in\mathbb{C}} \sum_{i\in\mathbb{I}} \sum_{j\in\mathbb{I}} Z_{c,i} Z_{k,j} \left(M_{c,k}^{\mathrm{R}} - M_{i,j}^{\mathrm{S}}\right)^2 \tag{S5}$$

for some $\alpha \in [0,1]$. From this perspective, the GW distance component of Eq. (S5) can capture the spatial relations between spots. In the following, we show how our spatial distance function d_{S} defined in Eq. (4) is related to this distance function for a particular choice of metrics $\mathbf{M}^{\mathrm{R}}$ and $\mathbf{M}^{\mathrm{S}}$.

Proposition 1. *Let $\beta = \sum_{i\in\mathbb{I}} \sum_{j\in\mathbb{I}} (1 - M_{i,j}^{\mathrm{S}})^2 p_i p_j$. Assuming that $\mathbf{M}^{\mathrm{R}}$ is a discrete metric so that $M_{c,c}^{\mathrm{R}} = 0$ and $M_{c,k}^{\mathrm{R}} = 1$, for $c, k \in \mathbb{C}$, $c \neq k$, then*

$$\mathrm{GW}(\mathbf{Z}) = \beta + \sum\nolimits_{i\in\mathbb{I}} \sum\nolimits_{j\in\mathbb{I}} \left(2M_{i,j}^{\mathrm{S}} - 1\right) \langle \mathbf{Z}_{:,i}, \mathbf{Z}_{:,j} \rangle$$

Proof. Given $M_{c,k}^{\mathrm{R}} = 1$ for $c \neq k$ and $M_{c,c}^{\mathrm{R}} = 0$, we obtain

$$\begin{aligned}
\mathrm{GW}(\mathbf{Z}) =& \sum_{i\in\mathbb{I}} \sum_{j\in\mathbb{I}} \sum_{c\in\mathbb{C}} \left(M_{i,j}^{\mathrm{S}}\right)^2 Z_{c,i} Z_{c,j} + \sum_{i\in\mathbb{I}} \sum_{j\in\mathbb{I}} \sum_{c\in\mathbb{C}} \sum_{k\in\mathbb{C}, k\neq c} \left(1 - M_{i,j}^{\mathrm{S}}\right)^2 Z_{c,i} Z_{k,j} \\
=& \sum_{i\in\mathbb{I}} \sum_{j\in\mathbb{I}} \sum_{c\in\mathbb{C}} \left(\left(M_{i,j}^{\mathrm{S}}\right)^2 - \left(1 - M_{i,j}^{\mathrm{S}}\right)^2\right) Z_{c,i} Z_{c,j} \\
&+ \sum_{i\in\mathbb{I}} \sum_{j\in\mathbb{I}} \sum_{c\in\mathbb{C}} \sum_{k\in\mathbb{C}} \left(1 - M_{i,j}^{\mathrm{S}}\right)^2 Z_{c,i} Z_{k,j} \\
=& \sum_{i\in\mathbb{I}} \sum_{j\in\mathbb{I}} \left(2M_{i,j}^{\mathrm{S}} - 1\right) \langle \mathbf{Z}_{:,i}, \mathbf{Z}_{:,j} \rangle + \beta,
\end{aligned}$$

where we have used $\beta = \sum\limits_{i\in\mathbb{I}} \sum\limits_{j\in\mathbb{I}} \left(1 - M_{i,j}^{\mathrm{S}}\right)^2 \sum\limits_{c\in\mathbb{C}} \sum\limits_{k\in\mathbb{C}} Z_{c,i} Z_{k,j} = \sum\limits_{i\in\mathbb{I}} \sum\limits_{j\in\mathbb{I}} \left(1 - M_{i,j}^{\mathrm{S}}\right)^2 p_i p_j$ since $\sum\limits_{c\in\mathbb{C}} Z_{c,i} = p_i$ and $\sum\limits_{k\in\mathbb{C}} Z_{k,j} = p_j$. □

Observe that $\langle \mathbf{Z}_{:,i}, \mathbf{Z}_{:,j} \rangle$ measures similarity between composition of spots i and j. Consequently, for a discrete metric $\mathbf{M}^{\mathrm{R}}$ (i.e., when cell populations are radically different), minimizing $\mathrm{GW}(\mathbf{Z})$ encourages spots i and j to acquire similar compositions when $2M_{i,j}^{\mathrm{S}} - 1 > 0$, discourages spots i and j from acquiring similar compositions when $2M_{i,j}^{\mathrm{S}} - 1 < 0$, and is indifferent to the composition of spots i and j when $2M_{i,j}^{\mathrm{S}} - 1 = 0$.

To produce a metric $\mathbf{M}^{\mathrm{S}}$ that captures the dissimilarity of spots in terms of their locations and expressions, we define $D_{i,j}^{\mathrm{L}}$ and $D_{i,j}^{\mathrm{E}}$ to represent distance of spots (i, j)

with respect to their locations and expressions, respectively

$$D^{\mathrm{L}}_{i,j} = \mathbf{1}_{\mathrm{condition}} \left(\|\mathbf{x}_i - \mathbf{x}_j\| > \bar{d} \right)$$
$$D^{\mathrm{E}}_{i,j} = \mathrm{d}_{\cos} \left(\mathbf{X}^{\mathrm{S}}_{i,:}, \mathbf{X}^{\mathrm{S}}_{j,:} \right) ,$$

where $\bar{d}$ is a given distance threshold, and $D^{\mathrm{E}}_{i,j}$ is computed with respect to all genes in S (i.e., $\mathbb{G}^{\mathrm{S}}$). Finally, we take $\mathbf{M}^{\mathrm{S}}$ to be the average of $\mathbf{D}^{\mathrm{L}}$ and $\mathbf{D}^{\mathrm{E}}$:

$$\mathbf{M}^{\mathrm{S}} = (\mathbf{D}^{\mathrm{L}} + \mathbf{D}^{\mathrm{E}})/2 \tag{S6}$$

Remark 1. $\mathbf{M}^{\mathrm{S}}$ *is a metric in the domain of S, since both* $\mathbf{D}^{\mathrm{L}}$ *and* $\mathbf{D}^{\mathrm{E}}$ *are metrics.*
Remark 2. *With the definition of* $\mathbf{M}^{\mathrm{S}}$ *in Eq.* (S6) *and* $\mathbf{M}^{\mathrm{R}}$ *a discrete metric,* $GW(\mathbf{Z})$ *encourages adjacent spots to attain similar compositions if their expressions are similar, (ii) discourages distant spots from attaining similar compositions if their expressions are different, and (iii) is indifferent to pair* (i, j) *when* i *and* j *are distant or different in expressions, but not both.*

From this perspective, our spatial distance function $\mathrm{d_S}$ defined in Eq. (4) specializes $\mathrm{GW}(\mathbf{Z})$ to encouraging adjacent spots to attain similar compositions if their expressions are similar. Note that our definition of the set of spatial pairs $\mathbb{P}$ given in Eq. (10) uses the same distance threshold $\bar{d}$. However, given the non-convex and quadratic nature of $\mathrm{GW}(\mathbf{Z})$, our $\mathrm{d_S}$ distance function is computationally more appealing as it is convex and scales linearly with the number of spots.

Supplementary Figures

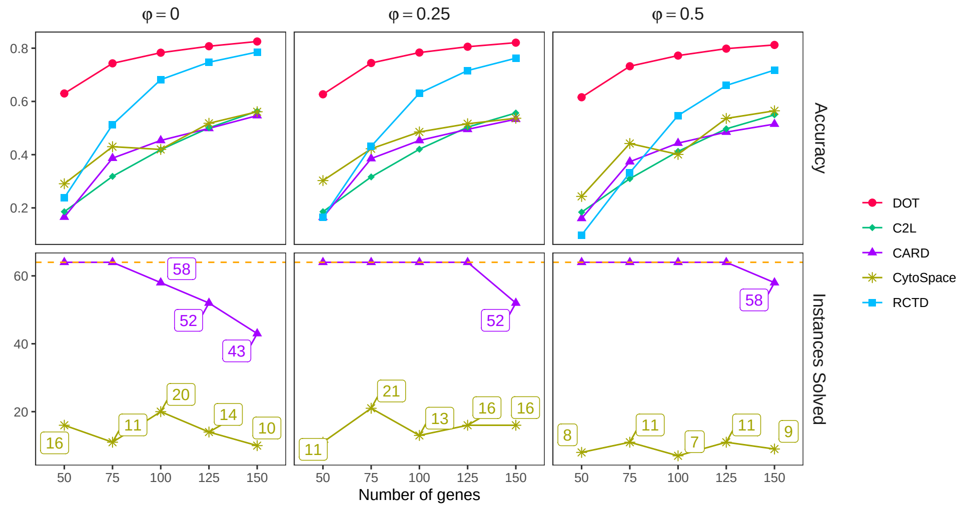

Supplementary Fig. 1: Performance of different methods in transferring cell types to spatial locations in high-resolution spatial data as function of the gene coverage in the spatial data (x-axis) and as function of different amounts of noise in gene expression (φ). First row shows accuracy values representing the median of 64 values. Second row illustrates the number of instances (out of 64) solved by each model. All methods except `CytoSpace` and `CARD` solved all 64 instances across all experiments. (Supplementary data for Fig. 2) Source data are provided as a Source Data file.

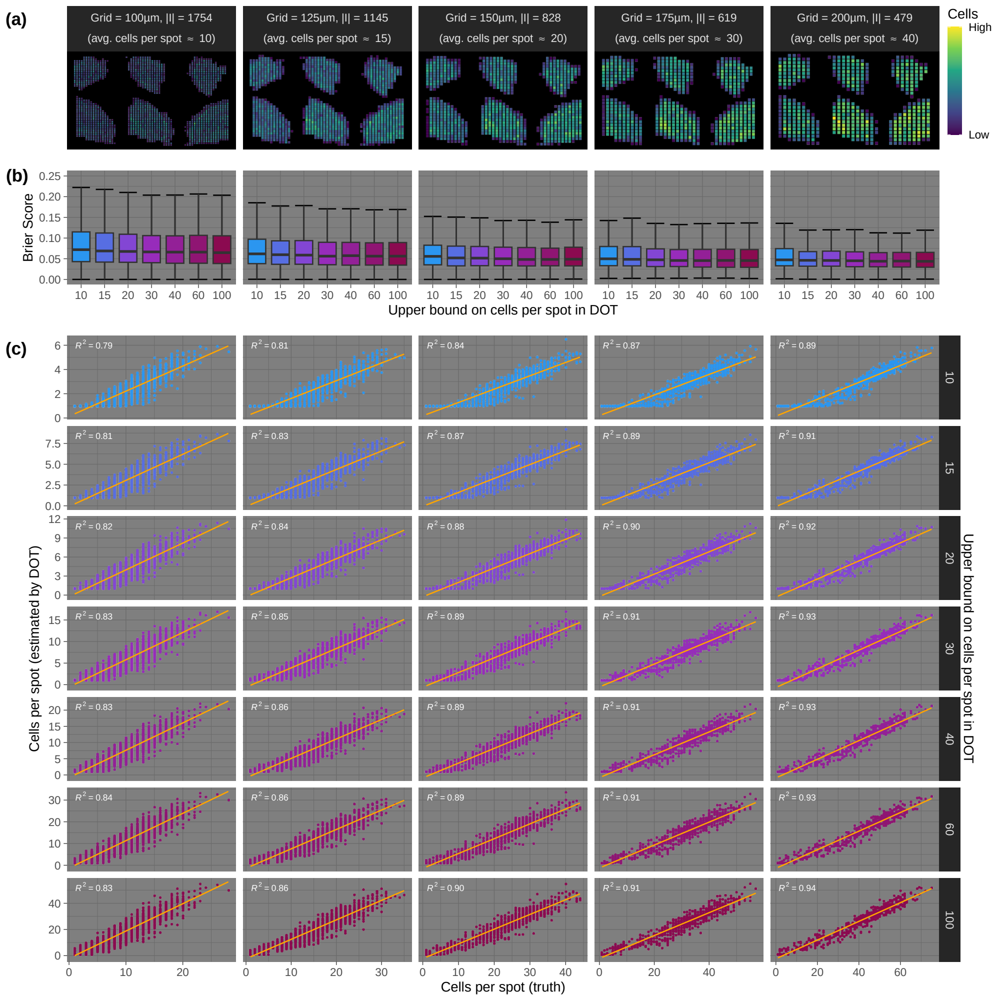

Supplementary Fig. 2: Robustness of DOT under different spatial resolutions and different choices for DOT's upper bound parameter on the number of cells per spot (i.e., n; Results). **a** The first six samples of the MERFISH MOp data are used for these experiments. Each cell from the MERFISH data is randomly assigned to a cell of the same 'subclass' from the scRNA-seq data. The assigned cells are then aggregated at five levels of grid length (100 µm, 125 µm, . . . , 200 µm) to form multicell spots (see Methods for more details). The grid lengths correspond to a total of $|I|$ =1754, 1145, 828, 619 and 479 spots and approximately 10, 15, 20, 30, and 40 cells per spot on average, respectively. **b** Sensitivity of performance of DOT on decomposing the multicell spots into cell types as a function of spatial resolution (i.e., grid length and average number of cells per spot) and the upper bound parameter n, with $n \in \{10, 15, 20, 30, 40, 60, 100\}$. generally improves as resolution decreases (grid length increases), but, for a fixed spatial resolution, it remains unaffected by the choice of parameter n. **c** The number of cells per spot as estimated by DOT exhibits a strong correlation with the ground truth (i.e., number of cells within each grid) for different choices of n at different resolutions even when n is smaller than the expected number of cells per spot. Source data are provided as a Source Data file.

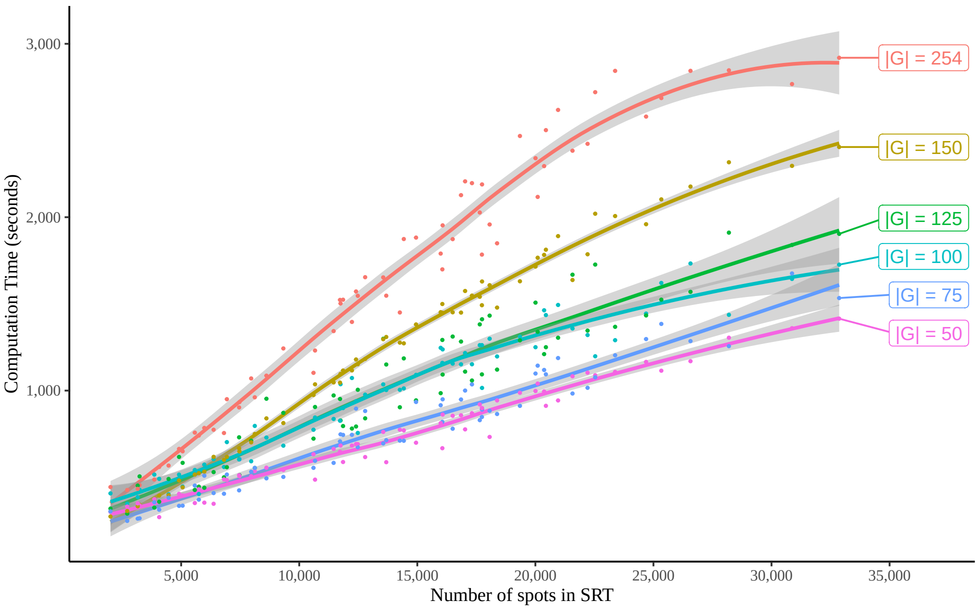

Supplementary Fig. 3: Total computation time of DOT for instances with different numbers of cells (spots) and genes in SRT. MERFISH MOp slides are categorized into 12 batches (according to mouse/sample ids) and incrementally aggregated within each batch to create spatial data with the number of spots ranging between 2,000 and 33,000 spots. Source data are provided as a Source Data file.

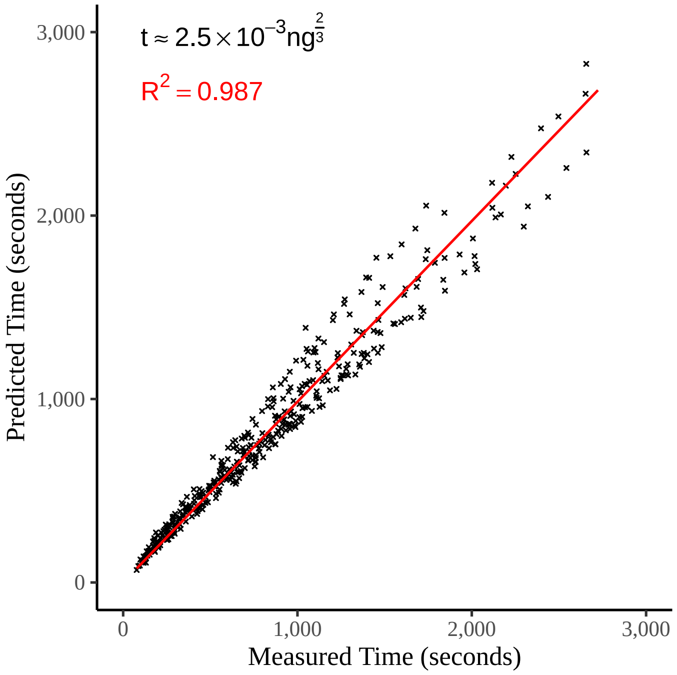

Supplementary Fig. 4: Computation time of DOT as a function of number of spots (n) and number of genes (g) for the same experiments described in Supplementary Fig. 3. Source data are provided as a Source Data file.

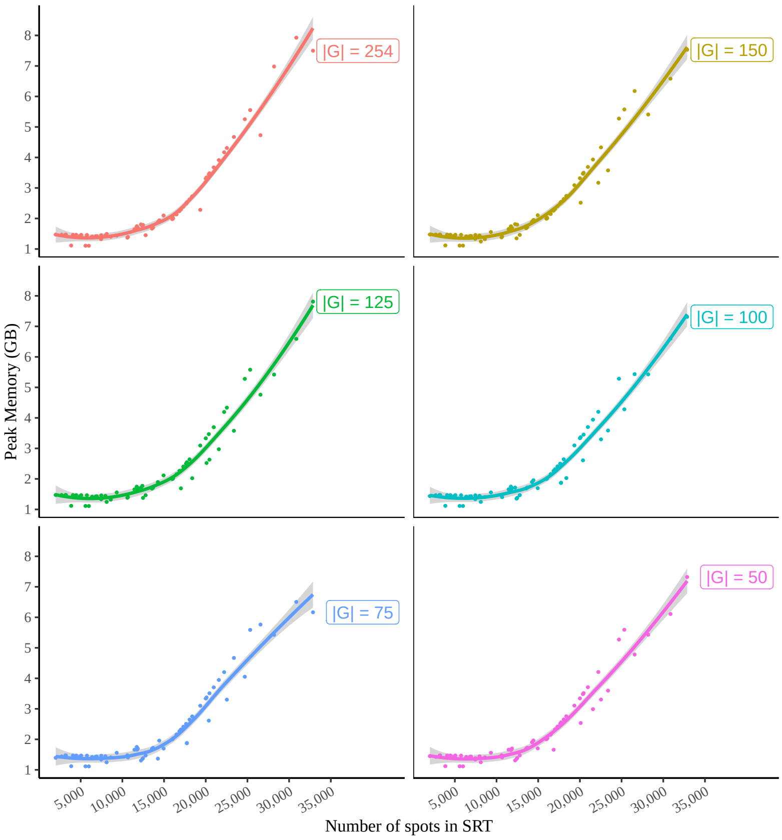

Supplementary Fig. 5: Peak memory usage of DOT for instances of different sizes for the same experiments described in Supplementary Fig. 3. Source data are provided as a Source Data file.

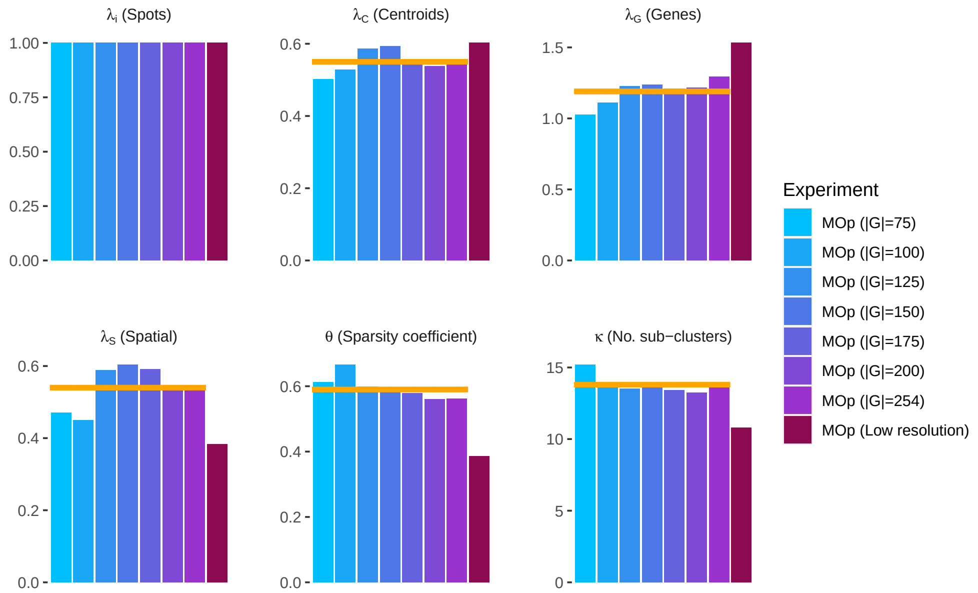

Supplementary Fig. 6: Top performing parameter settings for the simulated low resolution MOp and high resolution MOp with different numbers of genes. The first six samples of the MERFISH MOp data are used for these experiments. Synthetic low resolution data from the same samples are created as elaborated in Methods. Parameters are selected from $\lambda_C \in \{0, 0.25, 0.5, 1\}$, $\lambda_S \in \{0, 0.25, 0.5, 1\}$, $\lambda_G \in \{0.5, 1, 2\}$, $\theta \in \{0, 0.25, 0.5, 1\}$, and $\kappa \in \{1, 10, 20\}$, and the average of the parameters over the top 10% combinations are reported for each number of genes. Source data are provided as a Source Data file.

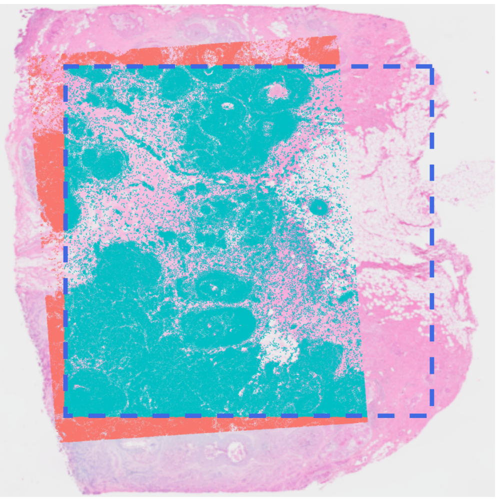

Supplementary Fig. 7: Common region (cyan) in the capture areas of Visium (dashed blue lines) and Xenium (dark orange) in human breast cancer. The pink region is the H&E image accompanying Visium.

Supplementary Notes

In this section, we provide additional notes on the datasets used in this study.

Mouse Primary Motor Cortex (MOp)

We used the spatially resolved cell atlas of the MOp recently generated using multiplexed error-robust fluorescence in situ hybridization (MERFISH) technology and made publicly available by [25]. The processed dataset contains normalized RNA counts of 254 genes and coordinates of the boundaries of a total of 280,186 segmented cells across 64 samples in the MOp of two adult mice, with the number of cells within each sample ranging from 1000 to 7500 cells. We computed the (x, y) coordinates of the center of each cell by taking the average of the coordinates of its boundary. The study also identifies 99 trasncriptionally distinct cell types by community detection applied on a cell similarity graph. The clustering resulted in 39 excitatory neuronal cell types (clusters), 42 inhibitory neuronal cell types, 14 non-neuronal cell types, and four other cell types.

The corresponding scRNA-seq data comes from a cell atlas of the MOp [26]. We used the scRNA-seq dataset `scRNA_10X_v2_A`, which contains 145,748 cells and 100 cell types. After removing the unannotated cells and low quality cell types (as categorized in the study), we retrieved 124,330 cells and 90 distinct cell types. For computational efficiency, we also selected the top 5,000 variable genes according to their means and variances [27].

Mouse Primary Somatosensory Cortex (SSp)

Similar to MOp, another well-studied tissue area is the primary somatosensory cortex area (SSp). Here, we used high-resolution spatial data coming from the osmFISH platform [28], which contains measurements of 33 genes across 4,837 cells, as well as annotations based on 11 major cell types. For reference scRNA-seq data with matched cell types, we used the annotations independently generated by [29] using 5,392 single cells in the same SSp region.

Developing Human Heart

For the developing human heart, we used subcellular spatial data generated by the ISS platform [30], which contains tissue sections from human embryonic cardiac samples collected at different times. We selected the `PCW6.5` slide which contains measurements of 69 genes across 17,454 cells as well as annotations of 12 major cell types. The same

study also provides scRNA-seq data for a similar slide, which contains matched cell types for 3,253 cells.

Human Dorsolateral Prefrontal Cortex

Dorsolateral Prefrontal Cortex (DFLPC) is a brain area that has been implicated in a number of neuropsychiatric disorders. The DLPFC dataset coming from [31] contains spatial gene expression profiles of 12 Visium samples corresponding to two pairs of directly adjacent serial tissue sections from three independent neurotypical adult donors. Each samples contains between 3460 and 4789 spots, with the spots manually annotated with the six layers of the human DLPFC (L1 to L6) and white matter (WM).

Human Breast Cancer

Breast cancer is a complex disease with significant cellular and molecular heterogeneity. We used three different sets of human breast cancer datasets for our experiments.

For our cell-type deconvolution experiments on human breast cancer spatial transcriptomics (Results), we used Visium samples `1142243F`, `1160920F` and `CID44971` from [32], all of the triple negative breast cancer (TNBC) subtype. The samples contain 4665, 4846 and 1161 spots, respectively, and are manually annotated by a pathologist into 12 categories based on the respective H&E images. Additionally, we used Spatial Transcriptomics samples `G2` and `H1` from [33], all of the HER2+ subtype. These samples contain 467 and 613 spots, respectively, and are manually annotated by a pathologist with six labels based on the morphology of the associated H&E images. For both data sets, we used the scRNA-seq data from [32] to decompose the spots into nine major cell types with cancer-associated fibroblasts (CAFs) and perivascular-like (PVL) cells combined as Mesenchymal for visualization.

For our experiments on estimating the expression of unmeasured genes (Results), we used the spatial data from breast cancer tumor microenvironment produced by the 10X Xenium In Situ technology [34]. The dataset is unique in that it contains both high-resolution (Xenium) and low-resolution (Visium) spatial data of serial sections from the same tissue. The high-resolution data contains two replications produced by the recent 10X Xenium In Situ technology. We used `Xenium_FFPE_Human_Breast_Cancer_Rep1`, which contains the spatial information of 313 genes for 167,782 cells. The low-resolution spatial dataset is produced by the 10X Visium Spatial Transcriptomics technology, which contains the spatial information

of 18,000 genes for 4,992 multicell spots. The dataset also contains the dissociated scRNA-seq data coming from a tissue section adjacent to the tissue sections used for Visium and Xenium workflows. We used the `Single Cell Gene Expression Flex (FRP)` data which contains expression of 18,000 genes across 30,365 cells.

Supplementary Fig. 7 illustrates the common capture areas of Visium and Xenium tissues.

Supplementary References

[1] Jaggi, M., Lacoste-Julien, S.: On the global linear convergence of Frank-Wolfe optimization variants. Advances in Neural Information Processing Systems **28** (2015)

[2] Bertsekas, D.P.: Nonlinear Programming. Athena Scientific, Belmont, Massachusetts (2016)

[3] Wai, H.-T., *et al.*: Decentralized Frank–Wolfe algorithm for convex and nonconvex problems. IEEE Trans. Automat. Contr. **62**(11), 5522–5537 (2017)

[4] Jaggi, M.: Revisiting Frank-Wolfe: Projection-free sparse convex optimization. In: International Conference on Machine Learning, pp. 427–435 (2013). PMLR

[5] Zhang, Y., Li, B., Giannakis, G.B.: Accelerating Frank-Wolfe with weighted average gradients. In: ICASSP 2021-2021 IEEE International Conference on Acoustics, Speech and Signal Processing (ICASSP), pp. 5529–5533 (2021). IEEE

[6] Garber, D., Meshi, O.: Linear-memory and decomposition-invariant linearly convergent conditional gradient algorithm for structured polytopes. Advances in Neural Information Processing Systems **29** (2016)

[7] Villani, C.: Topics in Optimal Transportation vol. 58. American Mathematical Soc., Providence, Rhode Island (2021)

[8] Santambrogio, F.: Optimal Transport for applied mathematicians. Birkäuser, NY **55**(58-63), 94 (2015)

[9] Peyré, G., Cuturi, M., *et al.*: Computational Optimal Transport: With applications to data science. Found. Trends Mach. Learn. **11**(5-6), 355–607 (2019)

[10] Zhang, Z., Wang, M., Nehorai, A.: Optimal Transport in reproducing kernel

Hilbert spaces: Theory and applications. IEEE Transactions on Pattern Analysis and Machine Intelligence **42**(7), 1741–1754 (2019)

[11] Bunne, C., *et al.*: Learning generative models across incomparable spaces. In: International Conference on Machine Learning, pp. 851–861 (2019). PMLR

[12] Mialon, G., *et al.*: A trainable Optimal Transport embedding for feature aggregation. In: International Conference on Learning Representations (ICLR) (2020)

[13] Wang, W., et al.: Optimal transport for unsupervised denoising learning. IEEE Trans. Pattern Anal. Mach. Intell. (2022)

[14] Chuang, C.-Y., *et al.*: Measuring generalization with Optimal Transport. Advances in Neural Information Processing Systems **34**, 8294–8306 (2021)

[15] Titouan, V., *et al.*: Optimal Transport for structured data with application on graphs. In: International Conference on Machine Learning, pp. 6275–6284 (2019). PMLR

[16] Li, J., et al.: Divergence-agnostic unsupervised domain adaptation by adversarial attacks. IEEE Trans. Pattern Anal. Mach. Intell. (2021)

[17] Tong, A., *et al.*: TrajectoryNet: A dynamic Optimal Transport network for modeling cellular dynamics. In: International Conference on Machine Learning, pp. 9526–9536 (2020). PMLR

[18] Schiebinger, G., *et al.*: Optimal-transport analysis of single-cell gene expression identifies developmental trajectories in reprogramming. Cell **176**(4), 928–943 (2019)

[19] Forrow, A., Schiebinger, G.: LineageOT is a unified framework for lineage tracing and trajectory inference. Nat. Commun. **12**(1), 1–10 (2021)

[20] Nitzan, M., *et al.*: Gene expression cartography. Nature **576**(7785), 132–137 (2019)

[21] Cang, Z., Nie, Q.: Inferring spatial and signaling relationships between cells from single cell transcriptomic data. Nature Communications **11**(1), 2084 (2020)

[22] Zeira, R., *et al.*: Alignment and integration of spatial transcriptomics data. Nature

Methods **19**(5), 567–575 (2022)

[23] Klein, D., Palla, G., Lange, M., Klein, M., Piran, Z., Gander, M., Meng-Papaxanthos, L., Sterr, M., Bastidas-Ponce, A., Tarquis-Medina, M., et al.: Mapping cells through time and space with moscot. Preprint at http://biorxiv.org/content/10.1101/2023.05.11.540374 (2023)

[24] Mémoli, F.: Gromov–Wasserstein distances and the metric approach to object matching. Found. Comut. Math. **11**(4), 417–487 (2011)

[25] Zhang, M., Eichhorn, S.W., Zingg, B., Yao, Z., Cotter, K., Zeng, H., Dong, H., Zhuang, X.: Spatially resolved cell atlas of the mouse primary motor cortex by MERFISH. Nature **598**(7879), 137–143 (2021)

[26] Yao, Z., Liu, H., Xie, F., Fischer, S., Adkins, R.S., Aldridge, A.I., Ament, S.A., Bartlett, A., Behrens, M.M., Berge, K., *et al.*: A transcriptomic and epigenomic cell atlas of the mouse primary motor cortex. Nature **598**(7879), 103–110 (2021)

[27] Stuart, T., Butler, A., Hoffman, P., Hafemeister, C., Papalexi, E., Mauck, W.M., Hao, Y., Stoeckius, M., Smibert, P., Satija, R.: Comprehensive integration of single-cell data. Cell **177**(7), 1888–1902 (2019)

[28] Codeluppi, S., Borm, L.E., Zeisel, A., La Manno, G., Lunteren, J.A., Svensson, C.I., Linnarsson, S.: Spatial organization of the somatosensory cortex revealed by osmFISH. Nature Methods **15**(11), 932–935 (2018)

[29] Yao, Z., Van Velthoven, C.T., Nguyen, T.N., Goldy, J., Sedeno-Cortes, A.E., Baftizadeh, F., Bertagnolli, D., Casper, T., Chiang, M., Crichton, K., *et al.*: A taxonomy of transcriptomic cell types across the isocortex and hippocampal formation. Cell **184**(12), 3222–3241 (2021)

[30] Asp, M., Giacomello, S., Larsson, L., Wu, C., Fürth, D., Qian, X., Wärdell, E., Custodio, J., Reimegård, J., Salmén, F., *et al.*: A spatiotemporal organ-wide gene expression and cell atlas of the developing human heart. Cell **179**(7), 1647–1660 (2019)

[31] Maynard, K.R., Collado-Torres, L., Weber, L.M., Uytingco, C., Barry, B.K., Williams, S.R., Catallini, J.L., Tran, M.N., Besich, Z., Tippani, M., *et al.*: Transcriptome-scale spatial gene expression in the human dorsolateral prefrontal

cortex. Nature Neuroscience **24**(3), 425–436 (2021)

[32] Wu, S.Z., Al-Eryani, G., Roden, D.L., Junankar, S., Harvey, K., Andersson, A., Thennavan, A., Wang, C., Torpy, J.R., Bartonicek, N., *et al.*: A single-cell and spatially resolved atlas of human breast cancers. Nature Genetics **53**(9), 1334–1347 (2021)

[33] Andersson, A., Larsson, L., Stenbeck, L., Salmén, F., Ehinger, A., Wu, S.Z., Al-Eryani, G., Roden, D., Swarbrick, A., Borg, Å., *et al.*: Spatial deconvolution of HER2-positive breast cancer delineates tumor-associated cell type interactions. Nature Communications **12**(1), 6012 (2021)

[34] Janesick, A., Shelansky, R., Gottscho, A.D., Wagner, F., Williams, S.R., Rouault, M., Beliakoff, G., Morrison, C.A., Oliveira, M.F., Sicherman, J.T., *et al.*: High resolution mapping of the tumor microenvironment using integrated single-cell, spatial and in situ analysis. Nature Communications **14**(1), 8353 (2023)
